# A novel single-portal arthroscopic technique for the management of pediatric humeral lateral condylar fractures

**DOI:** 10.3389/fped.2026.1751163

**Published:** 2026-01-22

**Authors:** Mingjing Li, Fan Li, Yushun Fang, Ming Tang, Jiang Xiang, Chunquan Zhu, Jian Xu, Zonghui Dai, Sen Tang, Fucheng Ouyang, Jiawen Yu

**Affiliations:** 1 Department of Pediatric Orthopedics, Wuhan Fourth Hospital, Wuhan, China; 2 Department of Sports Medicine, Wuhan Fourth Hospital, Wuhan, China

**Keywords:** arthroscopy, children, closed reduction, lateral humeral condyle fracture, proximal anterolateral portal

## Abstract

**Background:**

Surgical intervention is indicated for significantly displaced lateral humeral condyle fractures (LHCFs) in children. Arthroscopic-assisted closed reduction represents a minimally invasive alternative; however, its widespread adoption has been limited by the technical challenges inherent in both pediatric fracture management and elbow arthroscopy. This study introduces a simplified technique utilizing a single proximal anterolateral portal for arthroscopic-assisted reduction, which has shown promising efficacy and safety.

**Methods:**

A retrospective analysis was performed on 18 pediatric patients with LHCFs who underwent arthroscopic-assisted closed reduction via a single proximal anterolateral portal at our institution between March 2024 and February 2025. The cohort included 14 boys and 4 girls, with a mean age of 6.1 ± 1.6 years. The mean interval from injury to surgery was 4.7 ± 2.1 days. Data on fracture classification, operative time, duration of K-wire fixation, and functional outcomes were collected and analyzed.

**Results:**

All 18 patients successfully underwent the procedure. The mean operative time was 56.9 ± 10.0 min, and K-wires were maintained for a mean of 35 ± 8.5 days. At the final follow-up, no significant differences in the carrying angle were observed between the injured and contralateral limbs. According to the Flynn criteria, 16 cases were rated as excellent and 2 as good. One case of a superficial pin site infection resolved with conservative wound care. No instances of delayed union, nonunion, neurovascular injury, or compartment syndrome were recorded.

**Conclusion:**

The single proximal anterolateral portal technique for arthroscopic-assisted reduction of LHCFs facilitates minimally invasive debridement of the fracture site and provides direct visualization of the reduction process. This approach serves as a viable and effective alternative for managing lateral condylar fractures that are not amenable to conventional closed reduction due to severe displacement or a prolonged delay from injury. The technique demonstrates a favorable safety profile, and shows promise for broader clinical adoption pending further validation.

## Introduction

Lateral humeral condyle fracture (LHCF) represents one of the most common elbow fractures in children, accounting for approximately 12%–20% of pediatric upper limb fractures, second only to supracondylar humeral fractures (1). Surgical intervention is typically indicated when fracture displacement exceeds 2 mm (2). The conventional treatment involves open reduction through a lateral elbow approach combined with percutaneous pin fixation, which generally yields satisfactory outcomes (3). However, in response to growing aesthetic concerns among children and parents, as well as the need to preserve the local blood supply to the lateral humeral condyle, closed reduction and internal fixation has emerged as a viable alternative (4). Several studies have reported favorable clinical and radiographic results with closed reduction and internal fixation for these fractures (4–6). Current closed reduction techniques often rely on continuous intraoperative fluoroscopy, arthrography, or dynamic ultrasound guidance (5–7). However, in cases with marked displacement or delayed presentation, interposed hematoma or granulation tissue between fracture fragments may impede reduction. Since conventional closed methods cannot address such interposition, conversion to open reduction is frequently required (5–8).

Arthroscopically assisted closed reduction offers distinct advantages over both open surgery and conventional closed techniques for LHCFs (9). This minimally invasive approach enables direct visualization of the articular surface, debridement of the fracture site, and is applicable to a variety of fracture patterns (10, 11). Nevertheless, due to the technical challenges associated with pediatric elbow arthroscopy and fracture management, published experience with this technique remains limited. The confined intra-articular space in young children and the proximity of neurovascular structures further contribute to a steep learning curve (12). In this report, we introduce a single proximal anterolateral portal for elbow arthroscopy, which utilizes the brachioradialis muscle as a protective barrier for the radial nerve, thereby minimizing the risk of nerve injury. This approach enables effective fracture site debridement and facilitates closed reduction. In this study, we retrospectively analyzed the clinical data of 18 children with LHCFs treated with this technique in our pediatric orthopedics department between March 2024 and February 2025. We aim to evaluate its efficacy, describe the surgical steps, and assess its safety, to provide a new perspective on the minimally invasive management of pediatric LHCFs.

## Patients and methods

### Case inclusion and exclusion criteria

Inclusion Criteria: (1) Pediatric patients (≤14 years) diagnosed with a LHCF; (2) Time from injury to surgery ≤3 weeks; (3) Treatment involving arthroscopically-assisted closed reduction and percutaneous K-wire fixation via a single proximal anterolateral portal; (4) A complete postoperative follow-up of ≥6 months, including comprehensive clinical and radiographic data.

Exclusion Criteria: (1) Concurrent fractures in other parts of the ipsilateral limb; (2) Pathological fractures; (3) Open fractures; (4) Postoperative follow-up of less than 6 months.

Demographic information, injury mechanism, fracture classification, and the presence of any associated neurovascular injuries were retrieved from the hospital database. This study was approved by the Institutional Ethics Committee of Wuhan Fourth Hospital (Approval No. KY2025-098-01).

### Surgical technique

**Figure 1:**
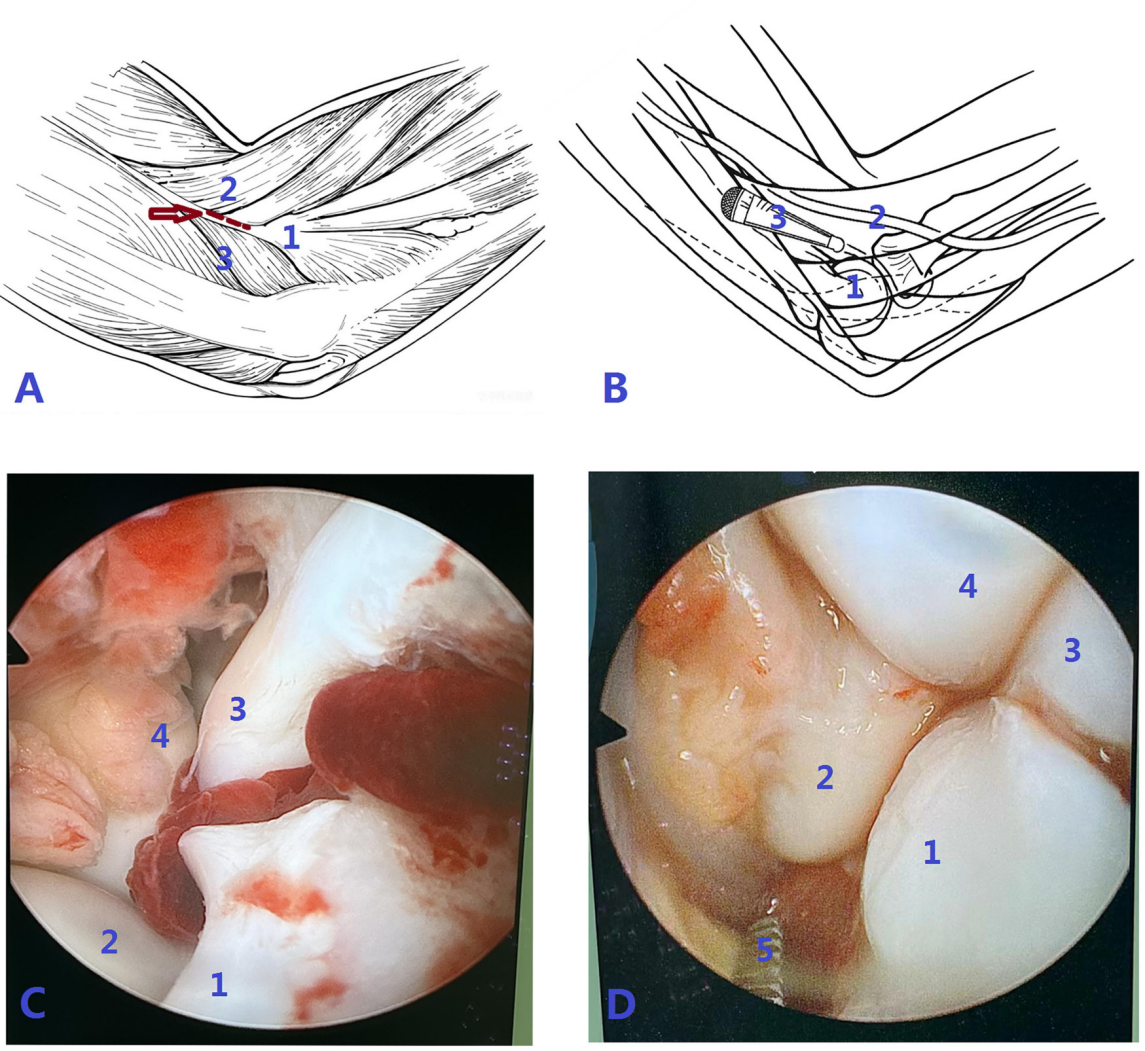
**(A,B)** are schematic diagrams illustrating the surgical procedure. A:The arrow indicates the surgical approach, represented by a dotted line (1. Lateral humeral condyle, 2. Brachioradialis, 3. Triceps brachii). **(B)** Arthroscope entering the elbow joint anterior to the lateral humeral condyle (1. Lateral humeral condyle, 2. Radial nerve, 3. Arthroscope). **C** and **D** are arthroscopic view of the elbow joint. **(C)** Fracture of the left lateral humeral condyle with hematoma visible between the fragments (1 Lateral humeral condyle; 2 Radial head; 3 Humeral trochlea; 4 Coronoid process of the ulna). **(D)** Image after debridement of the fracture site (1 Lateral humeral condyle; 2 Radial head; 3 Humeral trochlea; 4 Coronoid process of the ulna; 5 mosquito forceps).

**Figure 2:**
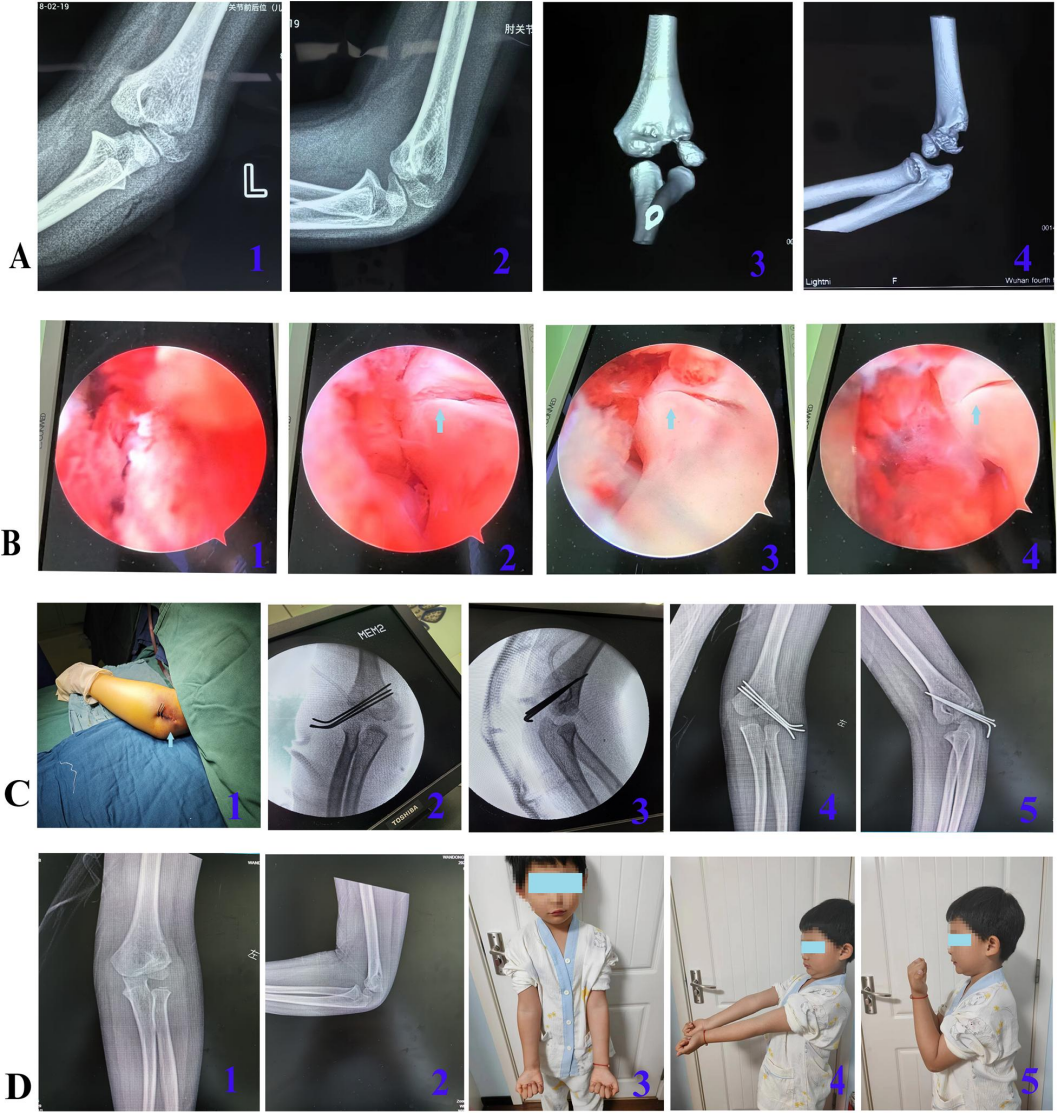
Representative case 1. A 6-year-and-7-month-old boy with a left lateral humeral condyle fracture caused by a fall. **A**: Preoperative imaging, including x-rays (1, 2) and CT scans (3, 4), showing a Jakob type III fracture. **B**: Intraoperative arthroscopic views: 1, extensive soft tissue interposition at the fracture site; 2, after debridement to relieve the interposition and preliminary reduction, converting the fracture to a Jakob type II; 3 and 4 show the post-reduction views, revealing an even articular surface and a fracture gap of <2 mm. **C**: 1, Postoperative appearance of the local incision; 2 and 3, intraoperative fluoroscopy images; 4 and 5, x-ray images at 1 month postoperatively. **D**: 1 and 2, x-ray images at 3 months postoperatively, showing fracture union; 3, 4, and 5, functional photographs at 6 months postoperatively, demonstrating no significant difference in the appearance and range of motion of the bilateral elbow joints.

**Figure 3:**
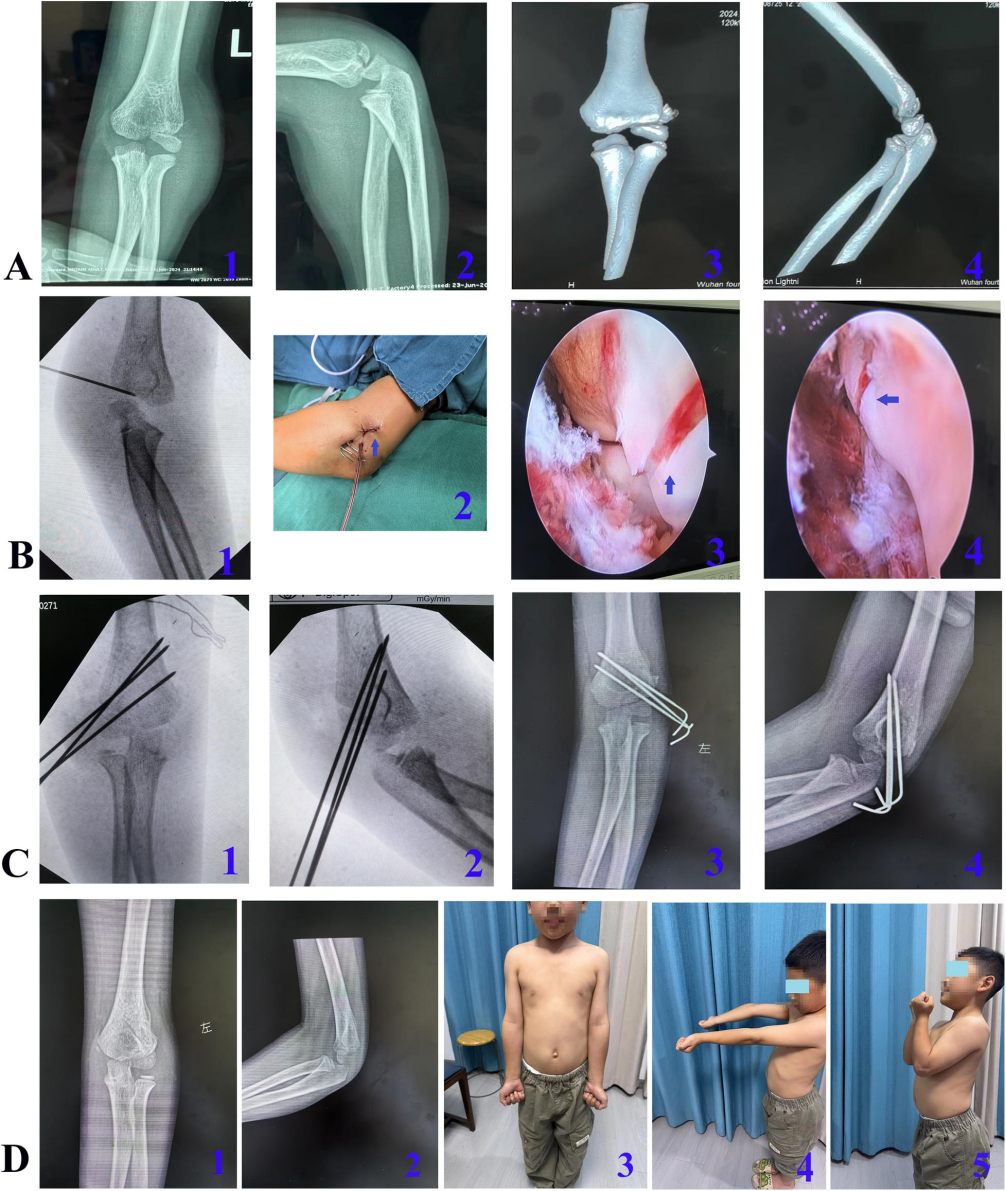
Representative case 2. A 7-year-and-6-month-old boy with a left lateral humeral condyle fracture caused by a fall. **A**: Preoperative imaging, including x-rays (1, 2) and CT scans (3, 4), showing a Jakob type II fracture. **B**: 1, Fluoroscopic localization of the fracture Line due to elbow swelling; 2, postoperative appearance of the local incision; 3 and 4, arthroscopic views before and after reduction, showing restoration of an even articular surface and a fracture gap of <2 mm. **C**: 1 and 2, intraoperative fluoroscopy images; 3 and 4, x-ray images at 1 month postoperatively. **D**: 1 and 2, x-ray images at 3 months postoperatively, showing fracture union; 3, 4, and 5, functional photographs at 6 months postoperatively, demonstrating no significant difference in the appearance of the bilateral elbow joints, with a 7° loss of extension in the affected elbow compared to the healthy side.

(1) The patient was positioned supine. Following the induction of general anesthesia or brachial plexus block, the affected limb was routinely disinfected, draped, and a sterile tourniquet was applied. (2) A single proximal anterolateral portal was established approximately 1 cm proximal and 1 cm anterior to the metaphyseal fracture line, through a ∼1.5 cm skin incision (Figure 1A). In cases of severe limb swelling obscuring anatomical landmarks, fluoroscopy was utilized to confirm the metaphyseal fracture location. For Jakob type II fractures, an arthroscopic cannula with a blunt trocar was introduced through this portal and advanced along the anterior humeral surface, followed by insertion of a 4.0 mm 30° arthroscope (Figure 1B). For Jakob type III fractures, the displaced fragment was first reduced using vascular forceps or a small periosteal elevator via this same portal to convert it to a Jakob type II fracture, after which the arthroscope was inserted. Continuous saline irrigation was employed to evacuate the intra-articular hematoma, allowing for visualization of the lateral humeral condyle fracture site and assessment for any associated injuries (Figure 1C). Through the same portal, mosquito forceps was used to clear blood clots or debris from the fracture site and joint space. Arthroscopic confirmation was obtained to ensure no soft tissue interposition at the fracture ends (Figure 1D). (3) With the elbow placed in a flexed position, an assistant maintained the arthroscope for visualization. The surgeon then reduced the fracture fragment using thumb pressure on the lateral condyle while stabilizing the forearm with the other hand. Sagittal angulation was corrected by adjusting elbow flexion/extension, and rotational displacement was addressed by rotating the forearm. Under continuous arthroscopic visualization confirming satisfactory fracture alignment and a congruent articular surface, the arthroscope was temporarily withdrawn. Percutaneous fixation was then achieved by driving two or three 1.5-mm K-wires into the lateral humeral condyle. The arthroscope was reinserted to verify the reduction. If a persistent articular step-off was observed, the K-wires were partially withdrawn, reduction was repeated, and the wires were re-advanced until an anatomical reduction was confirmed. The K-wires were then advanced to engage the opposite cortex. Satisfactory fracture reduction and correct K-wire positioning were confirmed by intraoperative fluoroscopy. (4) The K-wires were cut and left protruding percutaneously, the wound was irrigated and sutured, and the limb was immobilized in a long-arm posterior splint. If anatomical reduction could not be achieved arthroscopically, the procedure was converted to open reduction and internal fixation by extending the proximal anterolateral approach distally. Representative cases are shown in Figures 2, 3.

### Postoperative management and follow-up

The affected elbow was immobilized in a long-arm posterior splint for 4–8 weeks, depending on radiographic evidence of fracture healing. The percutaneous K-wires were removed in the outpatient clinic after confirmation of fracture union. All patients were encouraged to initiate and progressively increase active elbow range-of-motion exercises. Scheduled follow-up assessments were conducted at 4, 8, and 12 weeks postoperatively, with anteroposterior and lateral elbow radiographs obtained to monitor fracture healing. Final evaluations of elbow function and appearance were performed via outpatient consultation or WeChat review at 6–15 months after surgery.

The operative time, timing of K-wire removal, the carrying angle of both the affected and contralateral elbows, and the range of flexion and extension motion were recorded. Functional and cosmetic outcomes of the affected elbow were assessed using the Flynn's criteria (13). Complications were documented, including compartment syndrome, infection, neurovascular injury, avascular necrosis of the humeral head, delayed union, nonunion, and malunion.

### Statistical analysis

Quantitative data are presented as mean ± standard deviation ( x ± s). All statistical analyses were performed using IBM SPSS Statistics, Version 18 (SPSS Inc., Chicago, IL, USA). Comparisons of paired measurements between the affected and contralateral limbs were performed using paired-sample *t*-tests. Categorical data were compared using the Chi-square test or Fisher's exact test, as appropriate. A two-tailed *P*-value of < 0.05 was considered statistically significant. Given the exploratory nature of this study, no power analysis was performed.

## Results

From March 2024 to February 2025, a total of 18 children diagnosed with lateral condylar fractures of the humerus were enrolled in this study. Patient demographics and clinical outcomes are summarized in Table 1. The cohort included 14 boys and 4 girls, with a mean age of 6.1 ± 1.6 years (range: 3 years 1 month to 9 years 7 months). The time from injury to surgery ranged from 2 to 10 days, with a mean interval of 4.7 ± 2.1 days. All patients sustained injuries due to accidental falls and presented with no associated injuries. According to the Jakob classification, there were 13 type II and 5 type III fractures. Based on the Milch classification, there were 4 type I and 14 type II fractures. In all cases, disruption of the articular surface was observed, accompanied by evident hematoma or soft tissue interposition within the fracture gap.

**Table 1:** Demographics and outcomes of children with LHCFs treated with arthroscopy-assisted closed reduction.

Variables frequency (%) or mean ± SD (range)	Initial 6 month period	Subsequent 6 month period	Total
Case count	7	11	18
Age(yr)	6.4 ± 2.2 (3.1–9.6)	5.9 ± 1.1 (3.8–7.7)	6.1 ± 1.6 (3.1–9.6)
Sex			
Boy	7	7	14
Girl	0	4	4
Laterality			
Right	4	5	9
Left	3	6	9
Jakob classification			
Type Ⅱ	5 (71%)	8 (73%)	13 (72%)
Type Ⅲ	2 (29%)	3 (27%)	5 (28%)
Milch classification			
Type Ⅰ	2 (29%)	2 (18%)	4 (22%)
Type Ⅱ	5 (71%)	9 (82%)	14 (78%)
Trauma-to-surgery interval(d)	4.6 ± 2.0 (2–8)	4.7 ± 2.2 (2–10)	4.7 ± 2.1 (2–10)
Duration of surgery (min)	56.4 ± 10.3 (40–70)	57.3 ± 10.3 (45–80)	56.9 ± 10.0 (40–80)
Follow-up duration (mo)	10.9 ± 1.5 (9–13)	7.7 ± 1.0 (6–9)	8.9 ± 2.0 (6–13)
K-wire fixation period (d)	35.3 ± 3.9 (32–40)	35.1 ± 10.7 (27–63)	35.2 ± 8.5 (27–63)
ROM of elbow(°)	134.1 ± 2.4	133.6 ± 2.9	133.8 ± 2.7
Flynn criteria results			
Excellent	6	10	16
Good	1	1	2
Moderate	0		0
Poor	0		0
Complications			
Introgenic nerve injury	0	0	0
Infections	0	1	1
Nonunion/malunion	0	0	0
Lateral spur formation	3	7	10
Avascular necrosis	0	0	0

LHCFs: lateral humeral condyle fractures; ROM: range of motion.

All patients successfully underwent arthroscopically assisted closed reduction and percutaneous pinning via a single proximal anterolateral approach. Fracture fixation was achieved using two K-wires in 3 patients and three K-wires in the remaining 15. The incision length was approximately 1.5 cm. The mean operative time was 56.9 ± 10.0 min (range: 40–80 min). K-wires were removed at an average of 35 ± 8.5 days postoperatively, and all fractures progressed to union. The mean follow-up period was 8.9 ± 2.0 months (range: 6–13 months).

At the final follow-up, no significant differences in carrying angle were observed between the affected and contralateral limbs. One patient exhibited a 7° loss of extension, while two other patients showed losses of flexion of 5° and 8°, respectively, compared with the unaffected side. Based on the Flynn criteria, outcomes were rated as excellent in 16 patients and good in 2. Patients were divided into two groups based on the date of surgery (first vs. second six-month period), and no obvious trends were observed between the groups in terms of fracture type, operative time, time to K-wire removal, or functional outcomes. The most common radiological finding was the formation of lateral bone spurs, which were asymptomatic in all cases. Parents of 10 children reported prominence over the lateral aspect of the elbow, though no functional limitations were noted. One patient developed a pin-site infection at 3 weeks postoperatively, which resolved with wound care and early K-wire removal at 4 weeks. No other complications—such as iatrogenic nerve injury, compartment syndrome, delayed union, nonunion, or avascular necrosis of the humeral head—were observed during the follow-up period.

## Discussion

Lateral humeral condylar fractures are common pediatric injuries. Significantly displaced fractures often require surgical intervention to prevent complications such as malunion or nonunion (14). The traditional approach involves a lateral elbow incision to directly visualize the articular surface, debride the fracture ends, and achieve anatomical reduction followed by fixation with K-wires or screws, which has proven effective (1). However, the classic lateral incision results in a visible scar and extensive lateral soft tissue dissection may potentially affect the development of the lateral condyle (2). With advancements in minimally invasive techniques, various closed reduction methods are now available for treating these fractures. These techniques achieve satisfactory reduction while significantly improving cosmetic outcomes (5).

Studies indicate that fractures with 2–5 mm of displacement can often be successfully managed with closed reduction and internal fixation alone (5–7). For fractures with greater displacement, where the cartilaginous hinge is disrupted, intraoperative arthrography, ultrasound guidance, or arthroscopy can assist in assessing articular alignment. However, these methods carry a risk of misinterpretation. Temporin and Kang et al. have observed cases where routine radiographs showed displacement of <2 mm, yet arthroscopy revealed significant articular incongruity (11, 15). Compared to arthrography and ultrasound-guided techniques, arthroscopy allows direct visualization of the degree and direction of cartilaginous displacement, providing a more direct and accurate assessment of reduction quality.

Severely displaced LHCFs often exhibit significant hematoma or soft tissue interposition between fragments. It is well-established that relieving soft tissue interposition is crucial for achieving anatomical reduction. Multiple studies report that failed closed reduction under ultrasound or arthrography is primarily due to unresolved soft tissue interposition, necessitating conversion to open reduction (4, 7). Besides allowing direct visualization of cartilaginous reduction, arthroscopy offers the advantage of facilitating debridement between fracture fragments. As shown in Figure 1, arthroscopy provides a view comparable to that of the lateral elbow approach, with adjustable visualization of the anterior articular surface. It enables direct inspection of fracture ends, ensuring complete removal of intra-articular hematoma and interposed tissue. Arthroscopy-guided reduction allows dynamic monitoring of intraoperative reduction quality, reducing the need for fluoroscopy and minimizing potential iatrogenic damage caused by repeated K-wire penetration. This establishes a prognostic foundation for fracture healing and functional recovery unmatched by other reduction techniques. Compared to traditional open reduction, arthroscopy better preserves joint capsule integrity and has less impact on the blood supply of the lateral humeral condyle. With significantly smaller incisions, arthroscopic surgery can achieve comparable outcomes in fracture healing and functional recovery.

There are nine commonly used portal approaches in elbow arthroscopy, but there is no internationally standardized selection for treating lateral humeral condylar fractures (16). Perez and Weng et al. employed dual medial and lateral elbow portals for such fractures, with the advantage that the viewing and working portals do not interfere with each other, and the arthroscope does not hinder reduction during surgery (10, 17). Weng et al. reported that using dual medial-lateral portals achieved a nearly 50% success rate in early closed reduction of lateral humeral condylar fractures, which increased to 93% for procedures performed after about six months. They suggested that the learning curve for this technique is approximately six months. In young children, the limited working space of the elbow brings neurovascular structures closer to portal sites, increasing potential risks. To mitigate ulnar nerve injury, some authors opt for the safer proximal anterolateral portal combined with a direct lateral or proximal anteromedial portal (18). Additionally, hyperextension of the elbow should be avoided during surgery, as it may lead to transient radial nerve palsy.

In clinical practice, we have found that using a single proximal anterolateral approach is sufficient for visualization and debridement of the fracture site. This technique is relatively simple and easy to learn and master. Unlike degenerative or inflammatory conditions of the elbow, the elbow tissues in most children with lateral humeral condyle fractures are normal prior to injury. Therefore, there is no need to insert instruments such as shavers for debridement of cartilage or synovium, which is the main reason we adopt a single-portal approach. Secondly, incarcerated soft tissues between the fracture fragments can be easily removed with forceps without the need for additional portals, thereby minimizing trauma and avoiding potential iatrogenic nerve injuries associated with extra incisions. For significantly displaced lateral humeral condyle fractures, the lateral joint capsule is already ruptured. The proximal anterolateral approach is adjacent to the ruptured capsule, and we have found that it allows easy access into the joint intraoperatively without further damaging the capsule. Since this portal utilizes the brachioradialis muscle as a cushion to protect the radial nerve, and we perform the procedure with the patient in the supine position and the elbow flexed, radial nerve injury can be effectively avoided. Additionally, the arthroscopic view obtained through this approach is similar to the direct view in open reduction, which helps beginners become familiar with the arthroscopic perspective more quickly and facilitates guided closed reduction. There were no obvious trends in operative time or outcomes between the surgeries performed in the first six months and those in the latter six months. It appears to have a manageable learning curve based on operative time stability. Therefore, compared to the dual-portal arthroscopic-assisted technique, the single proximal anterolateral approach is potentially less resource-intensive and technically straightforward.

With the development of various closed reduction techniques, this method can now be applied to LHCFs with varying degrees of displacement. Some scholars have reported no difference in the distribution of fracture types between the closed reduction and open reduction groups (19). One study involving 39 pediatric patients with lateral humeral condyle fractures treated with arthroscopic assistance reported a closed reduction success rate approaching 77%, with Song type V fractures accounting for nearly one-fourth of the cases (11). These results indicate that fracture type is not an indicator for choosing between closed or open reduction; rather, the technical details during surgery determine the success of closed reduction. Studies have found that for Jakob type III or Song type V fractures, severe limb swelling and fragment incarceration with rotation are the main reasons for closed reduction failure. Our approach is to first release the intra-articular hematoma through the proximal anterolateral portal, and use forceps or a periosteal elevator to preliminarily release the incarcerated soft tissues, converting the fracture to a Jakob type II or Song type III/IV fracture, before proceeding with arthroscopic examination. Although we have only treated 18 cases so far, including 5 cases of Jakob type III fractures, all were successfully reduced closed under arthroscopy using this method, and the operative time did not significantly increase compared to other fracture types.

Due to the narrow joint space in children's elbows, the arthroscope may collide with or obstruct reduction of the lateral condyle fragment during reduction and fixation. Similar to the technique described by Weng et al., we use intermittent arthroscopy to effectively avoid this issue (10). If the arthroscope hinders reduction, it can be temporarily withdrawn. After provisional fixation, the arthroscope is reinserted to assess reduction. Final fixation is performed only after satisfactory reduction is confirmed. Intermittent irrigation also helps avoid excessive intra-articular pressure and reduces the risk of fluid extravasation into surrounding soft tissues, thereby effectively preventing compartment syndrome. Of course, as with other studies using dual medial and lateral portals for arthroscopic closed reduction, the posterior joint surface cannot be visualized through this single portal. Studies suggest that acceptable reduction is achieved when there is no step-off on the anterior cartilage surface under arthroscopy, the fracture gap is <2 mm, and intraoperative fluoroscopy confirms no separation of the metaphyseal fragment (19). Consistent with these findings, all our closed reduction cases showed satisfactory limb appearance and functional outcomes during follow-up.

However, this study has several limitations. As a single-center retrospective study, this study primarily demonstrates feasibility and safety rather than comparative efficacy. The limited sample size and lack of a control group may reduce the persuasiveness of the findings. Additionally, the follow-up period is relatively short, and growth-related complications may manifest beyond the follow-up period. These shortcomings highlight key areas for future research. We plan to continue follow-up and conduct further multicenter clinical studies to compare fracture healing, functional outcomes, and complications among different surgical techniques.

## Conclusion

The single proximal anterolateral portal arthroscopic-assisted reduction technique for lateral humeral condylar fractures enables minimally invasive debridement of the fracture site and allows direct visualization of fracture reduction. This technology is effective in treating lateral condylar fractures of the humerus in children, demonstrating high safety and showing promising clinical application prospects, making it worthy of further exploration.

## Data Availability

The datasets presented in this study can be found in online repositories. The names of the repository/repositories and accession number(s) can be found in the article/Supplementary Material.
